# Reparation of Bone after Trephining

**Published:** 1890-09-13

**Authors:** 


### REPARATION OF BONE AFTER TREPHINING.

A robust, powerful man, aged 48, was, on the 16th of last July, accidentally struck on the forehead by a quoit weighing 2 lb., hurled with the full strength of another player from a distance of only two or three paces. He fell stunned, but regained consciousness in less than ten minutes, bleeding freely. With the assistance of his companions he walked to a neighbouring dispensary, and after a few hours' rest to his home. Three days afterwards he was admitted into the Provincial Hospital, where he remained till his discharge on November 8th. Three months later he applied at the Princess' Hospital at Madrid, where he was admitted, under the care of Dr. F. Rubio. He was found to have a circular cicatrix 2 cms. wide on the upper part of the frontal eminence, a little to the right of the middle line. There was no paralysis, but occasional slight involuntary tremors of the left leg, not unlike commencing Jacksonian epilepsy, with transient loss of consciousness occurring several times a-day. His character was completely changed during the last three or four months; from being active and cheerful he had become apathetic and morose, his expression stupid, and his speech thick and slow. Trephining was determined on, and performed on February 11th of the present year when Dr. Rubio removed a loose piece of bone lying on the first frontal convolution and pressing on the membranes and brain beneath. Since the bone had been too much damaged to permit of its re-application after Dr. MacEwen's method, Dr. Rubio decided on availing himself of the results of the experiments which Dr. Seen, of Chicago, had performed on dogs only, of ingrafting portions of decalcified and aseptically preserved bone from the tibia of a cow, which he had prepared beforehand and in anticipation of the opportunity of carrying out the procedure on the human subject for the first time.

The results of both the primary and subsequent operations were most successful. The very next day the patient's expression and mental condition were greatly improved; he was lively and spoke clearly and with normal rapidity. The tremors had ceased and the £ts of vertigo did not recur. By the seventeenth day cicatrization was complete, a number of hard points could be felt in the now firmly embedded pieces of engrafted bone tissue which it appeared would in time become ossified throughout.

We have much pleasure in noting within a few weeks a second brilliant achievement in surgery from Spain, a country which has, hitherto, been not unjustly considered as lagging in the rear of medical progress.

Dr. Seen's procedure is as follows:—The lamellæ of bone are macerated in a 5 per cent. solution of hydrochloric acid, frequently renewed until the earthy matter is entirely removed. Pieces some millimeters in thickness, are then treated for forty-eight hours with a 2 per cent. solution of caustic potash or soda, in order to neutralize the acid. They are then stored in a 2 per 1,000 alcoholic solution of carrosive sublimate, until required for use, when they are to be dipped in a 5 per cent. aqueous solution of carbolic acid. Dr. Seen found that in nine operations on dogs he always considerably reduced, and in some instances completely closed the breach in the cranium by the formation of true bone.

